# GW501516, a PPARδ Agonist, Ameliorates Tubulointerstitial Inflammation in Proteinuric Kidney Disease via Inhibition of TAK1-NFκB Pathway in Mice

**DOI:** 10.1371/journal.pone.0025271

**Published:** 2011-09-22

**Authors:** Xu Yang, Shinji Kume, Yuki Tanaka, Keiji Isshiki, Shin-ichi Araki, Masami Chin-Kanasaki, Toshiro Sugimoto, Daisuke Koya, Masakazu Haneda, Takeshi Sugaya, Detian Li, Ping Han, Yoshihiko Nishio, Atsunori Kashiwagi, Hiroshi Maegawa, Takashi Uzu

**Affiliations:** 1 Department of Medicine, Shiga University of Medical Science, Otsu, Shiga, Japan; 2 Department of Medicine, Shengjing Hospital of China Medical University, Shenyang, LiaoNing, China; 3 Division of Endocrinology and Metabolism, Kanazawa Medical University, Kahoku-Gun, Ishikawa, Japan; 4 Department of Medicine, Asahikawa Medical College, Asahikawa, Hokkaido, Japan; 5 Nephrology and Hypertension, St. Marianna University School of Medicine, Kawasaki, Kanagawa, Japan; Institut National de la Santé et de la Recherche Médicale, France

## Abstract

Peroxisome proliferator-activated receptors (PPARs) are a nuclear receptor family of ligand-inducible transcription factors, which have three different isoforms: PPARα, δ and γ. It has been demonstrated that PPARα and γ agonists have renoprotective effects in proteinuric kidney diseases; however, the role of PPARδ agonists in kidney diseases remains unclear. Thus, we examined the renoprotective effect of GW501516, a PPARδ agonist, in a protein-overload mouse nephropathy model and identified its molecular mechanism. Mice fed with a control diet or GW501516-containing diet were intraperitoneally injected with free fatty acid (FFA)-bound albumin or PBS(−). In the control group, protein overload caused tubular damages, macrophage infiltration and increased mRNA expression of *MCP-1* and *TNFα*. These effects were prevented by GW501516 treatment. In proteinuric kidney diseases, excess exposure of proximal tubular cells to albumin, FFA bound to albumin or cytokines such as TNFα is detrimental. *In vitro* studies using cultured proximal tubular cells showed that GW501516 attenuated both TNFα- and FFA (palmitate)-induced, but not albumin-induced, *MCP-1* expression via direct inhibition of the TGF-β activated kinase 1 (TAK1)-NFκB pathway, a common downstream signaling pathway to TNFα receptor and toll-like receptor-4. In conclusion, we demonstrate that GW501516 has an anti-inflammatory effect in renal tubular cells and may serve as a therapeutic candidate to attenuate tubulointerstitial lesions in proteinuric kidney diseases.

## Introduction

Proteinuria is not only a clinical indicator of chronic kidney disease (CKD), but also a detrimental factor in progressive CKD [1], [2], [3]. In proteinuric kidney diseases, excessive albumin and several macromolecules bound to albumin, including free fatty acid (FFA), are filtered from glomeruli and reabsorbed into proximal tubular cells. This causes an inflammatory vicious cycle via secretion of pro-inflammatory cytokines, such as monocyte chemoattractant protein-1 (MCP-1), and subsequent infiltration of inflammatory cells, which secrete tumor necrotic factor-α (TNFα) [1], [2], [3], [4], [5]. Continuous inflammation triggers tubular cell damage, fibrosis and eventually progression to the final stage of kidney disease [5], [6]. Thus, suppression of inflammation caused by all these factors should serve as an important means to protect proximal tubular cells and maintain renal function in proteinuric kidney diseases.

Peroxisome proliferator-activated receptors (PPARs) are a nuclear receptor family of ligand-inducible transcription factors, composed of three different isoforms: PPARα, PPARδ and PPARγ [7], [8]. Acting as transcription factors, PPARs regulate various metabolic processes involving lipid metabolism, glucose homeostasis, cell differentiation and inflammation [7], [8], [9], [10], [11]. Recent reports demonstrated that PPARα and -γ agonists show renoprotective effects in a PPAR-dependent and independent manner [12], [13], [14], [15], [16], [17]; however, the physiological role and therapeutic potential of PPARδ in the kidney remain unclear. Several reports have shown that PPARδ agonists are potentially anti-inflammatory agents in some cell types and animal models [18], [19], [20], [21]. In summary, PPARδ agonists may show anti-inflammatory effects in renal proximal tubular cells and could be promising new therapeutic candidates for proteinuric kidney diseases.

To certify this hypothesis, we investigated whether GW501516, a highly selective PPARδ agonist [22], could attenuate tubulointerstitial lesions in a protein-overload mouse model (an established mouse model for studying interstitial lesions in proteinuric kidney diseases [4], [23]), and whether it could inhibit albumin-, saturated FFA (palmitate)- and TNFα-induced inflammation in cultured mouse proximal tubular cells. Our results showed that GW501516 exerted an anti-inflammatory effect in proximal tubular cells in both *in vivo* and *in vitro* studies, and it may be a promising new candidate for slowing down the progression of proteinuric kidney diseases.

## Results

### GW501516 attenuates interstitial inflammation and proximal tubular cell damage in a protein-overload mouse nephropathy model

**Figure 1:**
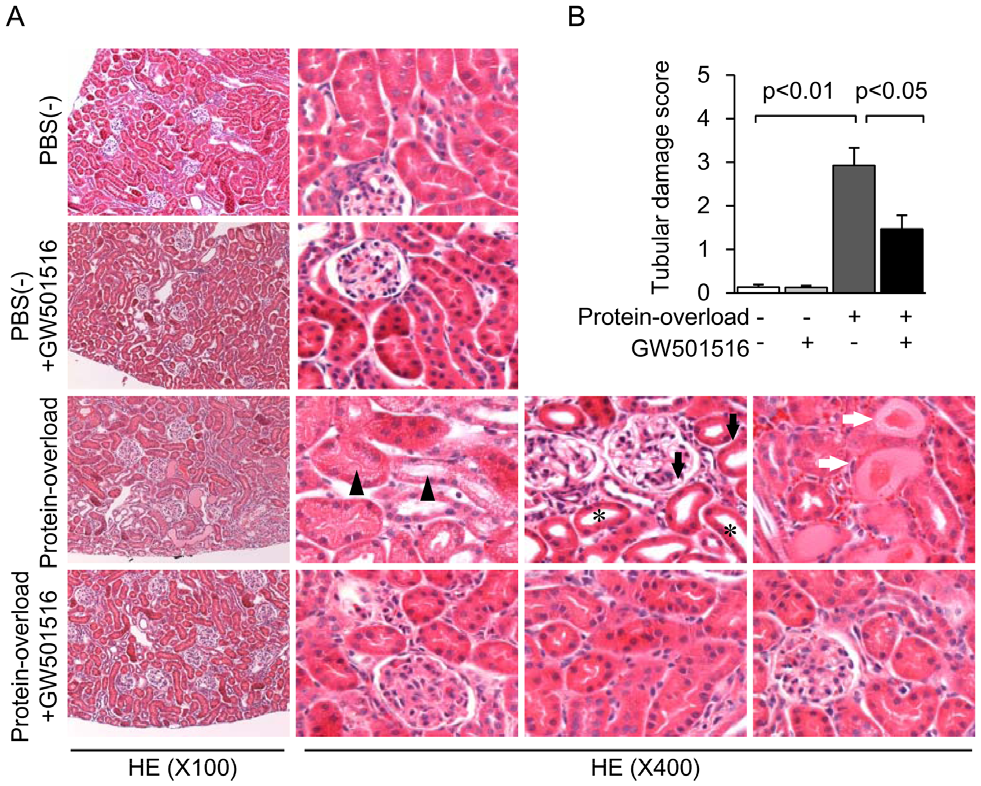
GW501516 attenuates renal proximal tubular cell damage in a protein-overload mouse renal injury model. (A) Representative HE staining of kidney sections from four groups [PBS(−), PBS(−) + GW501516, Protein-overload and Protein-overload + GW501516]. In the kidney of the protein-overload group, diffused tubular cell vacuolation (black triangles), tubular cell flattening (black arrows), tubular lumen dilation (asterisks) and cast formation (white arrows) were observed (magnification ×100 and ×400). (B) Tubulointerstitial damage scores of kidney sections from four groups. Data are shown as means ± SEM of each individual group.

To determine whether PPARδ agonists exert anti-inflammatory effects *in vivo*, we examined the effect of GW501516 on a protein-overload mouse model. First, we confirmed that bovine serum albumin (BSA) injection in mice increased urinary protein excretion, and that treatment with GW501516 did not affect basal and BSA-induced urinary protein excretion [(PBS(−) group: 3.26±0.66, PBS(−) + GW501516 group: 3.21±0.70, BSA group: 6.36±1.26, BSA + GW501516 group: 6.60±1.93 mg/day; data are shown as means ± SEM]. In mice fed with the control diet, intraperitoneal protein overload induced significant and severe tubular injury, characterized by diffused tubular cell vacuolation, tubular cell flattening, tubular lumen dilation and cast formation (Figure 1A and B). In the mice fed with the GW501516-containing diet, these tubulointerstitial damages were significantly attenuated (Figure 1A and B). The PBS(−) injection resulted in no apparent changes in proximal tubular cells of either control or GW501516-treated groups (Figure 1A and B).

**Figure 2:**
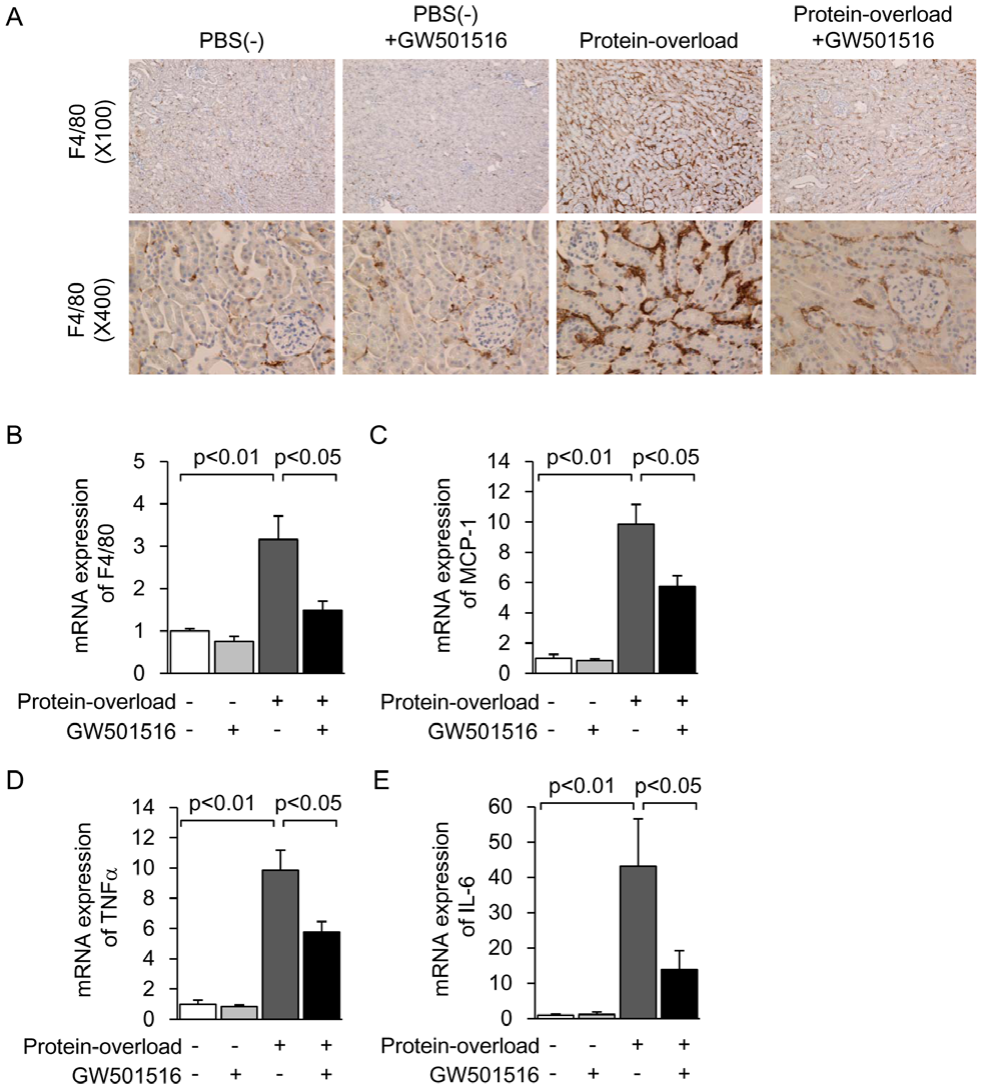
GW501516 suppresses interstitial inflammation in a protein-overload mouse renal injury model. (A) Representative photomicrographs of F4/80 immunostaining of the kidney from four groups [PBS(−), PBS(−) + GW501516, Protein-overload and Protein-overload + GW501516] (magnification, upper: ×100, lower: ×400). Renal mRNA expression of *F4/80* (B), *MCP-1* (C), *TNFα* (D) and *IL-6* (E) in kidney samples from the four groups. Data are shown as means ± SEM. Results are expressed as the fold change relative to the mean value of the mRNA expression level of the PBS(−) group. MCP-1: monocyte chemoattractant protein-1; TNFα: tumor necrotic factor α.

Protein overload induced the prominent infiltration of F4/80-positive macrophages into the renal interstitial area, which was markedly attenuated by GW501516 treatment (Figure 2A). Infiltration of F4/80-positive macrophages into glomeruli was not observed in the kidney of any group of mice (Figure 2A). The changes in *F4/80* expression were confirmed by mRNA levels, determined by real-time PCR (Figure 2B). Furthermore, protein overload significantly increased mRNA expression levels of inflammatory cytokines such as *MCP-1*, *TNFα and IL-6*. These increases were significantly attenuated by GW501516 treatment (Figure 2C, D and E). Protein overload and GW501516 treatment did not affect PPARδ expression levels in mice (data not shown). These results suggest that GW501516 shows an anti-inflammatory effect and protection of proximal tubular cells in the proteinuric kidney disease mouse model.

### GW501516 exerts anti-inflammatory effects in mouse cultured proximal tubular (mProx) cells

**Figure 3:**
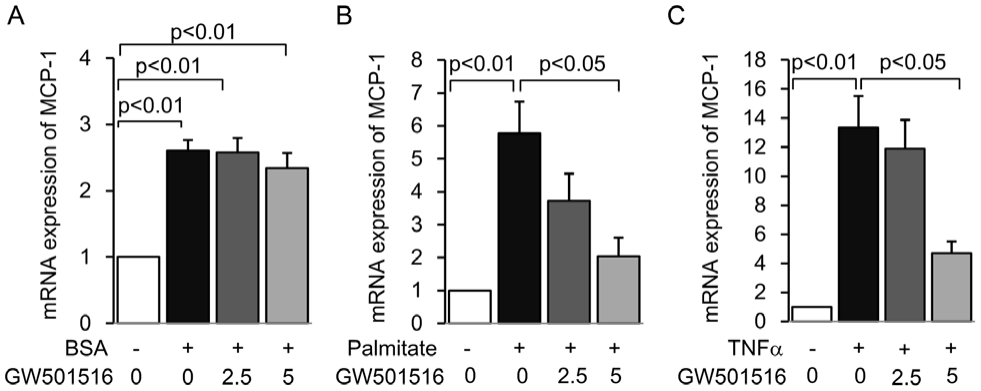
GW501516 inhibits palmitate- and TNFα-induced *MCP-1* expression in mouse proximal tubular (mProx) cells. mRNA expression of *MCP-1* determined by real-time PCR in cultured mProx cells stimulated by 30% BSA (A), 150 µM palmitate (B) or 10 nM of TNFα (C) for 12 hours, with or without a 3-hour pretreatment of different concentrations of GW501516 (0, 2.5, 5 µM of GW501516 dissolved in 0.05% DMSO). Results are expressed as fold change relative to control mRNA levels. Data are shown as means ± SEM of at least four independent experiments. MCP-1: monocyte chemoattractant protein-1; BSA: bovine serum albumin; TNFα: tumor necrotic factor α.

Because proximal tubular cells are the direct victims of overflowed protein [1], [2], [3], we examined the anti-inflammatory effect of GW501516 on cultured mouse proximal tubular cells, treated with pro-inflammatory molecules associated with proteinuria-induced inflammation, such as FFA-free albumin [24], [25], albumin-bound saturated FFA (palmitate) [26] and TNFα. GW501516 failed to inhibit FFA-free albumin-induced *MCP-1* expression (Figure 3A). However, GW501516 inhibited palmitate- and TNFα-induced increases in *MCP-1* mRNA expression in a dose-dependent manner (Figure 3B and C). These stimuli and treatment did not affect PPARδ expression levels (data not shown). These results suggest that the GW501516-mediated suppression of *MCP-1* expression is specific to palmitate and TNFα in cultured mProx cells.

### PPARδ is not required for GW501516-mediated anti-inflammatory effects in cultured proximal tubular (mProx) cells

**Figure 4:**
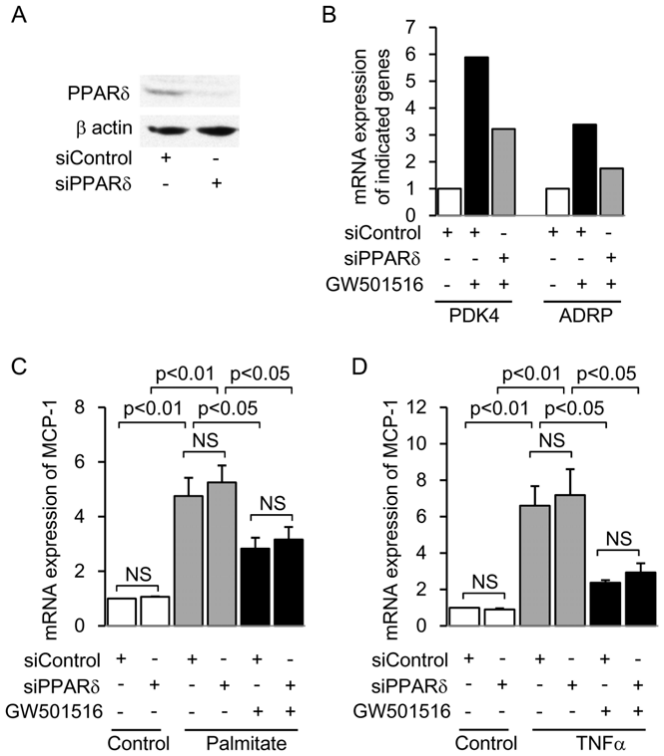
Knockdown of PPARδ gene expression does not affect GW501516-mediated inhibition of *MCP-1* expression in mouse proximal tubular (mProx) cells treated with palmitate or TNFα. (A) Representative immunoblot showing protein expression of PPARδ in siRNA-mediated PPARδ-knockdown and control cells. (B) mRNA expression of PPARδ-target genes (*PDK4* and *ADRP*) in PPARδ-knockdown and control cells treated with or without GW501516. Results are expressed as fold change relative to the mRNA from the control group. *PDK4*: Pyruvate dehydrogenase kinase 4; *ADRP*: Adipocyte differentiation-related protein. The effect of PPARδ knockdown on GW501516-mediated inhibition of palmitate- (C) and TNFα- (D) induced *MCP-1* expression. Results are expressed as fold change relative to the mRNA from the control group. Data are shown as means ± SEM of three independent experiments. MCP-1: monocyte chemoattractant protein-1; TNFα: tumor necrotic factor α.

**Figure 5:**
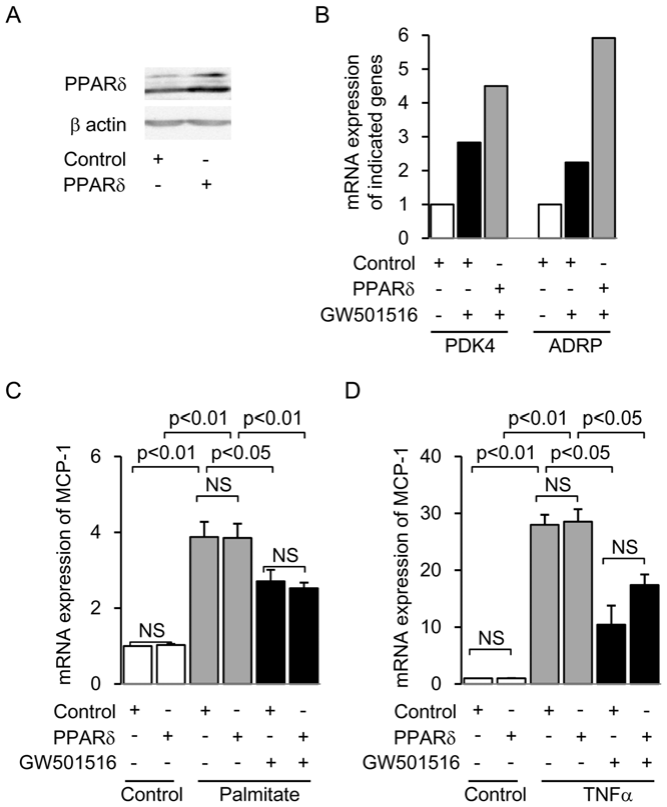
Overexpression of PPARδ does not affect GW501516-mediated attenuation of *MCP-1* expression in mouse proximal tubular (mProx) cells treated with palmitate or TNFα. (A) Representative immunoblot showing protein expression of PPARδ in retrovirus-mediated PPARδ-overexpressing cells and control cells. (B) mRNA expression of PPARδ-target genes (*PDK4* and *ADRP*) in PPARδ-overexpressing and control cells treated with or without GW501516. Results are expressed as fold change relative to the mRNA from the control group. *PDK4*: Pyruvate dehydrogenase kinase 4; *ADRP*: Adipocyte differentiation-related protein. The effect of PPARδ overexpression on GW501516-mediated inhibition of palmitate- (C) and TNFα- (D) induced *MCP-1* expression. Results are expressed as fold change relative to the mRNA from the control group. Data are shown as means ± SEM of four independent experiments. MCP-1: monocyte chemoattractant protein-1; TNFα: tumor necrotic factor α.

The molecular mechanism of PPAR agonist-mediated anti-inflammatory effects is cell-dependent [11], [27]. Also, some PPAR agonists have been shown to have an anti-inflammatory effect via direct inhibition of an intracellular signaling pathway, but not via ligand-activation of PPARs and subsequent upregulation of their target genes [11], [27]. Thus, we investigated whether PPARδ itself is required for the anti-inflammatory effect of GW501516 in cultured mProx cells, using siRNA-mediated PPARδ knockdown and retrovirus-mediated PPARδ overexpression. We confirmed the decreased PPARδ protein expression in siRNA-mediated PPARδ-knockdown mProx cells (Figure 4A). Furthermore, the mRNA level of PPARδ target genes was decreased in these cells (Figure 4B). GW501516 significantly attenuated both palmitate- and TNFα-induced *MCP-1* expression levels in mProx cells transfected with control siRNA (Figure 4C and D). This response was not affected by siRNA-mediated PPARδ knockdown (Figure 4C and D). Moreover, we generated retrovirus-mediated PPARδ-overexpressing mProx cells and confirmed the functional overexpression of PPARδ (Figure 5A and B). The GW501516 inhibitory effect on both palmitate- and TNFα-induced *MCP-1* expression was not enhanced in retrovirus-mediated PPARδ-overexpressing mProx cells (Figure 5C and D). Thus, neither knockdown nor overexpression of PPARδ changed the anti-inflammatory effect of GW501516. These results suggest that PPARδ itself is not essential for the anti-inflammatory effect of GW501516.

### GW501516 exerts its anti-inflammatory effect by inhibition of the TAK1-NFκB pathway

**Figure 6:**
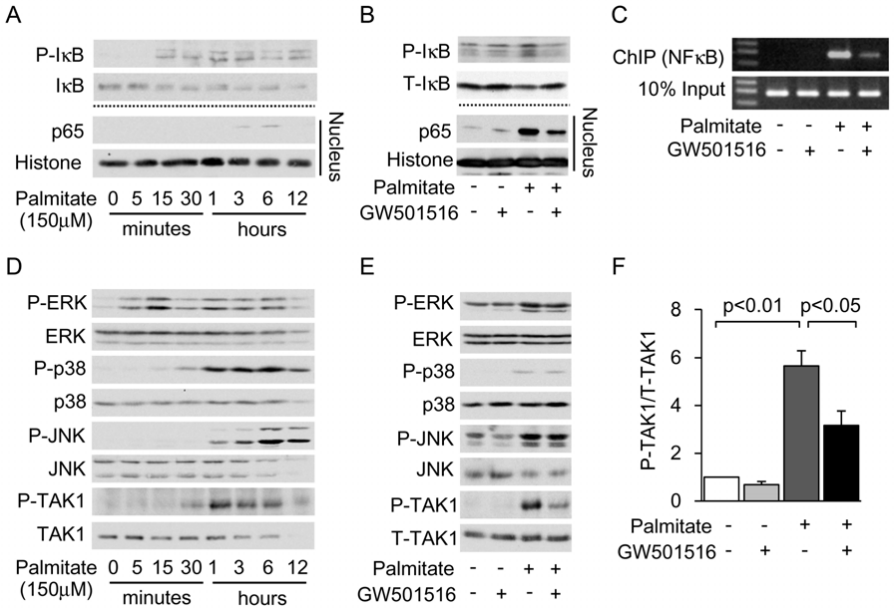
GW501516 inhibits palmitate-induced NFκB activation via inhibition of TAK1 in mouse proximal tubular (mProx) cells. (A) Immunoblot analysis showing palmitate-induced phosphorylation of IκB in the total cell lysate and nuclear translocation of the p65 subunit in a time course experiment. (B) Immunoblot analysis showing the effect of GW501516 on palmitate-induced phosphorylation of IκB and nuclear translocation of p65. The pretreatment time with GW501516 was 3 hours and the stimulation time with palmitate was 3 hours for phosphorylation of IκB and nuclear translocation of p65. The images are representative pictures from three independent experiments. (C) Effect of GW501516 on DNA binding of p65 to the *MCP-1* promoter determined by chromatin immunoprecipitation (ChIP) analysis in palmitate-treated mProx. (D) Immunoblot analysis showing palmitate-induced phosphorylation of MAPKs (P-ERK1/2, P-p38, P-JNK) and TAK1 in a time course experiment. (E) Immunoblot analysis showing the effect of GW501516 on phosphorylation of MAPKs and TAK1. Pre-incubation time with GW501516 was 3 hours, and stimulation time with palmitate was 1 hour. Images are representative figures from three independent experiments. TAK1: TGF-β activated kinase 1. (F) Quantitative results of the ratio of band density of P-TAK1 and T-TAK1.

We continued to elucidate the identity of the signaling molecules involved in the anti-inflammatory effect of GW501516, specifically focusing on signaling molecules associated with the NFκB pathway, which is a main inflammation regulatory pathway. In cultured mProx cells incubated with palmitate, phosphorylation of IκB and subsequent nuclear translocation of the p65 component of NFκB were observed after 1 hour of stimulation (Figure 6A). Palmitate-induced phosphorylation of IκB and nuclear translocation of p65 were attenuated by pretreatment with GW501516 (Figure 6B). Furthermore, we found that GW501516 inhibited palmitate-induced DNA binding of p65 to the *MCP-1* promoter, as determined by chromatin immunoprecipitation (ChIP) analysis (Figure 6C). Next, we checked the profiles of upstream and regulatory molecules associated with receptor-mediated NFκB activation, such as mitogen activated protein kinases (MAPKs; ERK, p38 and JNK) [28], [29] and TGF-β activated kinase 1 (TAK1) [30], [31]. The phosphorylation levels of the MAPKs and TAK1 were increased after 1 hour of palmitate stimulation (Figure 6D). GW501516 inhibited the palmitate-induced phosphorylation of TAK1 (Figure 6E and F), but not of the MAPKs (Figure 6E).

**Figure 7:**
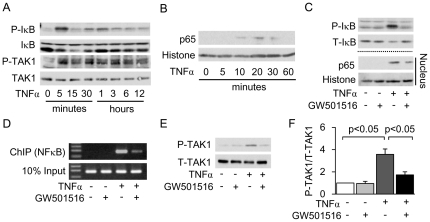
GW501516 inhibits TNFα-induced NFκB activation via inhibition of TAK1 in mouse proximal tubular (mProx) cells. Immunoblot analysis showing TNFα-induced phosphorylation of IκB and TAK1 (A) and nuclear translocation of the p65 subunit (B) in a time course experiment. (C) Immunoblot analysis showing the effect of GW501516 on TNFα-induced phosphorylation of IκB and nuclear translocation of p65. The pretreatment time with GW501516 was 3 hours and the stimulation time with TNFα was 5 minutes for phosphorylation of IκB and 10 minutes for nuclear translocation of p65. Images are representative results from three independent experiments. (D) Effect of GW501516 on DNA binding of p65 to the *MCP-1* promoter determined by chromatin immunoprecipitation (ChIP) analysis in TNFα-treated mProx cells. (E) Immunoblot analysis showing the effect of GW501516 on TAK1 phosphorylation. Pre-incubation time with GW501516 was 3 hours and stimulation time with TNFα was 5 minutes. (F) Quantitative result of the ratio of band density of P-TAK1 and T-TAK1. Images are representative figures from three independent experiments. TAK1: TGF-β activated kinase 1; TNFα: tumor necrotic factor α.

Furthermore, we observed similar results with cultured mProx cells incubated with TNFα. TNFα increased IκB phosphorylation and subsequent nuclear translocation of p65 after 5 minutes of stimulation (Figure 7A and 7B). These changes and DNA binding of p65 to the *MCP-1* promoter were significantly attenuated by pretreatment with GW501516 (Figure 7C and D, respectively). Finally, TAK1 phosphorylation was increased after 5 minutes of TNFα stimulation (Figure 7E and F), which was suppressed by pretreatment with GW501516 (Figure 7E and F).

**Figure 8:**
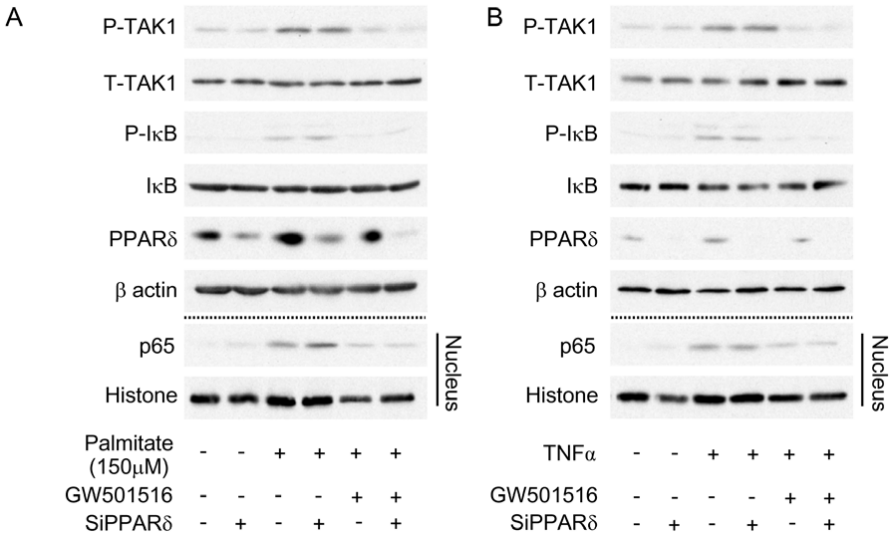
Knockdown of PPARδ gene expression does not affect the GW501516-mediated inhibitory effect on the TAK1-NFκB pathway in mouse proximal tubular (mProx) cells treated with palmitate or TNFα. Immunoblot analysis showing the effect of GW501516 on palmitate- (A) and TNFα- (B) induced phosphorylation of IκB and TAK1 in total cell lysate and nuclear translocation of the p65 subunit in siRNA-mediated PPARδ-knockdown and control cells. Images are representative figures from three independent experiments. TAK1: TGF-β activated kinase 1; TNFα: tumor necrotic factor α.

GW501516 inhibited palmitate- and TNFα-induced phosphorylation of TAK1 and IκB, and subsequent nuclear translocation of p65 even in mProx cells transfected with siRNA against PPARδ (Figure 8A and B), suggesting that GW501516 inhibited the TAK1-IκB pathway directly.

**Figure 9:**
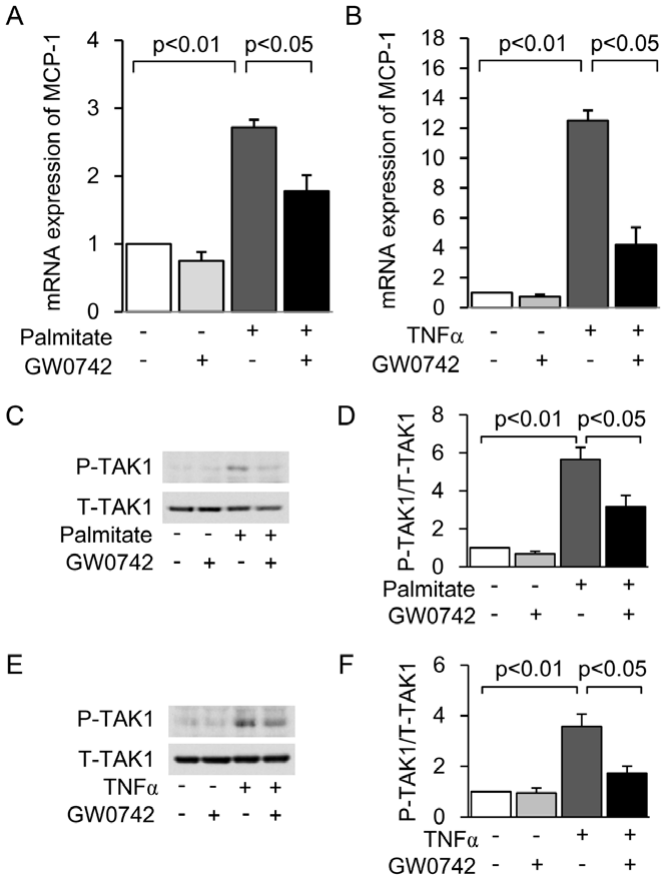
GW0742 inhibits palmitate- and TNFα-induced *MCP-1* expression as well as TAK1 phosphorylation in mouse proximal tubular (mProx) cells. mRNA expression of *MCP-1* determined by real-time PCR in cultured mProx cells stimulated by 150 µM palmitate (A) or 10 nM TNFα (B) for 12 hours, with or without a 3-hour pretreatment with GW0742 (5 µM GW0742 dissolved in 0.05% DMSO). Results are expressed as fold change relative to control mRNA levels. (C) Immunoblot analysis showing palmitate-induced phosphorylation of TAK1. (D) Quantitative result of the ratio of band density of P-TAK1 and T-TAK1. (E) Immunoblot analysis showing TNFα-induced phosphorylation of TAK1. (F) Quantitative result of the ratio of band density of P-TAK1 and T-TAK1. MCP-1: monocyte chemoattractant protein-1. TNFα: tumor necrotic factor α; TAK1: TGF-β activated kinase 1. Data are shown as means ± SEM of at least three independent experiments.

To investigate whether PPARδ agonist-mediated inhibition of TAK1 activation was specific to GW501516, we examined the anti-inflammatory effect and mechanism of another PPARδ agonist, GW0742, in mProx cells. Similarly to GW501516, GW0742 inhibited both palmitate- and TNFα-induced *MCP-1* expression (Figure 9A and B) and suppressed TAK1 phosphorylation (Figure 9C, D, E and F). These results suggest that PPARδ agonist-mediated inhibition of TAK1 activation is not specific to GW501516.

## Discussion

The present study shows that GW501516 attenuated tubulointerstitial lesions in a protein-overload nephropathy mouse model and that its renoprotective effect was achieved by its anti-inflammatory action in the kidney. These results highlight the possibility that GW501516 may be a novel therapeutic candidate for the improvement of renal prognosis in patients with proteinuric kidney diseases.

Growing evidence has demonstrated that PPARδ agonists have therapeutic potential against various disease models [18], [19], [20], [21], [22], [32], [33], [34]. Recent reports have shown that PPARδ agonists attenuated obesity-related diseases such as insulin resistance, diabetes and cardiovascular diseases, and regulated inflammatory cell function, which potentiated a systemic inflammatory response [18], [19], [20], [21], [22], [32], [33], [34]. Furthermore, Letavernier *et al.* showed the renoprotective effect of a PPARδ agonist in an ischemic reperfusion-mediated kidney injury [35]. In addition to these findings, we present evidence that PPARδ agonists have yet more therapeutic potential against proteinuric kidney diseases.

The protein-overload model is a well-established model for studying the renal tubulointerstitial lesions caused by proteinuria [4], [23]. In this model, upregulation of MCP-1 was demonstrated and the anti-*MCP-1* gene therapy attenuated interstitial lesions [36], which verified that attenuation of inflammation through inhibition of MCP-1 should serve as a therapeutic target in proteinuric kidney diseases. In our study, we confirmed that protein overload induced *MCP-1* expression and subsequent renal inflammation, and these inflammation-associated renal damages were attenuated by treatment with GW501516. To our knowledge, this is the first report showing that a pharmacological agent can improve tubulointerstitial lesions in this model.

**Figure 10:**
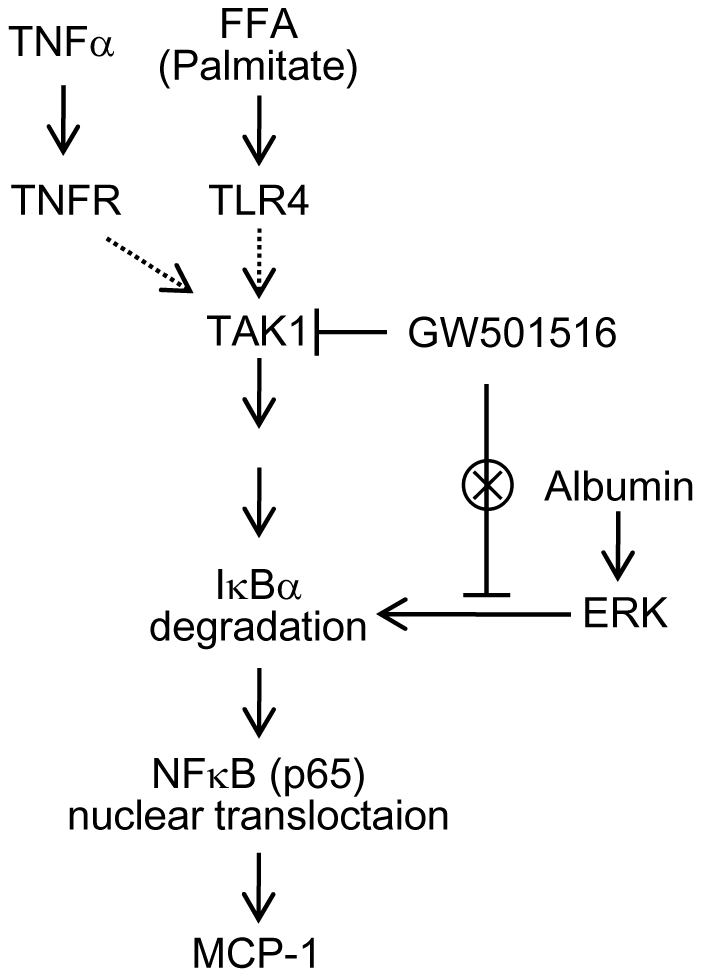
Proposed mechanism underlying the GW501516-mediated anti-inflammatory effect in proximal tubular cells in proteinuric kidney diseases. Both TNFα and palmitate induce the phosphorylation of TAK1 in a receptor dependent manner. The phosphorylation of TAK1 activates the NFκB pathway via degradation of IκB. The p65 subunit of NFκB translocates into the nucleus, upregulates the transcription of the pro-inflammatory gene, *MCP-1*, and causes inflammation. GW501516 can inhibit the phosphorylation of TAK1, but not of MAPKs (including ERK), and the activation of the NFκB-associated inflammatory response in proximal tubular cells. TNFα: tumor necrotic factor α; TNFR: TNFα receptor; TLR4: toll-like receptor 4; IKK: IκB Kinase; FFA: free fatty acid; MCP-1: monocyte chemoattractant protein-1.

Our *in vitro* study has identified the detailed molecular mechanism underlying the anti-inflammatory effect of GW501516 in the kidney. In proteinuric kidney diseases, albumin, FFAs bound to albumin and inflammatory cytokines coordinately mediate interstitial inflammation. In this study, we confirmed that all of these factors induced overexpression of *MCP-1* in cultured proximal tubular cells via NFκB activation. Interestingly, GW501516 attenuated *MCP-1* expression induced by palmitate and TNFα, but not albumin. These results suggest that the anti-inflammatory effect of GW501516 is not universal in NFκB-associated inflammatory processes; hence we focused on the upstream signaling to NFκB. NFκB activation is regulated by a variety of molecular pathways such as MAPKs and TAK1 [28], [29], [30]. Our group previously demonstrated that albumin-induced *MCP-1* expression was MAPK (ERK)-dependent [25]. Here we show that GW501516 could not attenuate albumin-induced *MCP-1* expression, suggesting that inhibition of ERK activity is not the target of the anti-inflammatory effect of GW501516. In contrast, GW501516 inhibited palmitate- and TNFα-induced *MCP-1* expression via the inhibition of TAK1 activation, without affecting MAPK activity. Furthermore, albumin failed to induce phosphorylation of TAK1 (data not shown). Thus, for the first time, we concluded that inhibition of TAK1, but not MAPKs, is involved in the mechanism underlying the anti-inflammatory effect of GW501516 (Figure 10). To our knowledge, few reports have shown the interaction between PPAR agonists and TAK1. Our current findings may enrich the knowledge of anti-inflammatory mechanisms of PPAR agonists.

To date, the mechanism underlying the anti-inflammatory effect of PPARδ agonists has been focused on BCL6 protein. The release of BCL6 from PPARδ is known to contribute to any of the anti-inflammatory actions of PPARδ agonists in macrophages, both *in vitro* [21], [37] and *in vivo* [21]. In other tissues it is not so clear; the inhibitory actions of PPARδ on adhesion molecules expression in endothelial cells have been reported to be both BCL6-dependent [38] and BCL6-independent [39], suggesting that the BCL6 theory is cell-specific and ligand-specific. We showed that suppression of *PPARδ* gene expression had no effect on *MCP-1* expression in mProx cells, indicating that BCL6 is not involved in the anti-inflammatory mechanism of GW501516 in proximal tubular cells.

Also in our study, we demonstrated that PPARδ itself is not essential for the anti-inflammatory effect of GW501516. The knockdown or overexpression of PPARδ could neither impede nor enhance the anti-inflammatory action of GW501516. GW501516 has been shown to exert an anti-inflammatory effect in many cell types [18], [19], [20], [21], but whether or not this effect is mediated through the PPARδ receptor has not been clarified. Although we demonstrated that two types of PPARδ agonists, GW501516 and GW0742, did not require PPARδ itself for their anti-inflammatory effects in cultured proximal tubular cells, it remains to be confirmed whether all PPARδ agonists function similarly, to conclude whether this mechanism is specific for GW501516 and GW0742 or global for PPARδ agonists. Furthermore, to elucidate whether GW501516 can improve renal inflammation in a PPARδ-independent manner *in vivo*, further examination of the effect of GW501516 on the progression of tubulointerstitial lesions induced by protein overload in PPARδ knockout mice is required.

In conclusion, we demonstrated that GW501516, a PPARδ agonist, showed a renoprotective effect by exerting an anti-inflammatory effect in proximal tubular cells, both *in vivo* and *in vitro*. Thus, it may be a promising candidate for slowing down the progression of chronic kidney diseases.

## Materials and Methods

### Antibodies and reagents

Antibodies were purchased from the following sources: TAK1 polyclonal antibody, phospho-TAK1 (T187), IκB-α, phospho-IκBα, p38-MAPK, phospho-p38-MAPK (Thr180/Tyr182), p44/42 MAPK, phospho-p44/42 MAPK, SAPK/JNK, phospho-SAPK/JNK (T183/Y185), Histone H3 and NFκB p65 were from Cell Signaling Technology (Beverly, MA, USA). Recombinant TNFα, sodium palmitate, albumin from bovine serum and GW0742 were purchased from Sigma (St. Louis, MO, USA). Small interference RNA against PPARδ was purchased from Applied Biosystems Japan, Ltd. GW501516 was from GlaxoSmithKline (London, UK).

### Animals

Animal care and treatment were performed in accordance with the guidelines of the Research Center for Animal Life Science of Shiga University of Medical Science (approval ID: 2010-2-13). Seven-week-old male C57BL/6 mice (CLEA Japan, Tokyo, Japan) were housed at a constant room temperature, 12-h dark/12-h light cycle, and freely fed a standard diet and tap water. Mice were randomly allocated to different groups and received therapeutic diet and treatment. The GW501516-containing rodent diet was made by evenly adding GW501516 to the control diet to a final concentration of 0.04% w/w. In the control diet, 10% of the total calories were from fat (5.5% from soybean oil and 4.5% from lard). All foods were purchased from Research Diets (New Brunswick, NJ, USA).

### Protein-overload mouse nephropathy model

Animal experiments were performed as described previously, with minor modifications [4], [23]. FFA-bound BSA was administered by intraperitoneal injection to induce renal tubulointerstitial damages. Mice were divided into the following four groups: PBS(−), PBS(−) + GW501516, protein-overload and protein-overload + GW501516 (n = 3, 4, 6 and 6, respectively). Mice were fed either a control diet or a GW501516-containing diet. Each group was given its diet for 10 days before starting treatments. Once a day for the following 4 days, the mice of each group were given an intraperitoneal injection of either 10 mg/g body weight FFA-bound BSA diluted in sterile PBS(−) or an equal volume of sterile PBS(−) as a control. On the fifth day, mice were anesthetized with intraperitoneal injection of pentobarbital sodium, then blood was drawn from the right atrium with a heparinized syringe and serum was separated for biochemical assays. Kidneys were removed for mRNA assays, histological examination and immunohistochemical staining, as previously reported [40], [41]. Urinary protein concentrations were measured with the Bradford method.

### Histological analyses

Fixed kidneys embedded in paraffin were sectioned to 3 µm thickness [40], [41]. Semiquantitative histological analysis of the tubular damage was performed using HE-stained sections from each mouse. Tubular damage scores, ranging from 0 to 4, were assessed according to both the severity and range of proximal tubular damages; an average was taken from the final score of 20 randomly selected, non-overlapping cortical areas under ×200 magnification [42]. Immunohistochemical staining was performed with the F4/80-specific monoclonal anti-rat antibody (Serotec, Oxford, UK), as previously described [40].

### Cell culture

Immortalized murine proximal tubular cells (mProx) were established as described previously [25]. First, we confirmed that the dosage of GW501516 used in the following experiments was not cytotoxic to the cells by using the AlamarBlue™ Assay (Alamar Bioscience, Sacramento, CA, USA; data not shown). Cells were cultured in DMEM containing 10% FBS, 100 U/ml penicillin and 100 µg/ml streptomycin at 37°C and 5% CO_2_ thermostat. Subconfluent cells were starved by incubation in 0.2% FCS DMEM for 9 h, then preincubated with GW501516 [dissolved in 10% dimethyl sulfoxide (DMSO)], at a final concentration of 2.5 and 5 µM, or 0.05% DMSO as control for 3 hours, followed by stimulation with 150 µM palmitate bound to 8.0% BSA for 12 h. We also performed the same experiment under stimulation with 10 nM TNFα and 30% BSA.

### Quantitative real-time PCR

Total RNAs were isolated from the kidney samples and cultured cells, and cDNAs were synthesized as previously reported [41]. The iQSYBR Green Supermix (Bio-Rad Laboratories, Hercules, CA, USA) was used for real-time PCR (ABI Prism TM 7500 Sequence Detection System; Perkin-Elmer Applied Biosystems, Foster City, CA, USA). The mRNA expression levels were quantified using the standard curve method [41]. Analytical data were normalized to the mRNA expression level of GAPDH as an internal control. Primer sequences are described in Table 1.

**Table 1:** Primer sequences used in this study.

Gene/Promoter	Forward	Reverse
*GAPDH*	ATGGCCTTCCGTGTTCCT	GCCTGCTTCACCACCTTCT
*MCP-1*	GCCCCACTCACCTGCTGCTACT	CCTGCTGCTGGTGATCCTCTTGT
*TNFα*	GACGTGGAACTGGCAGAAGAG	GCCACAAGCAGGAATGAGAAG
*PPARδ*	TAGAAGCCATCCAGGACACC	CCGTCTTCTTTAGCCACTGC
*F4/80*	CTTTGGCTATGGGCTTCCAGTC	GCAAGGAGGACAGAGTTTATCGTG
*PDK4*	TCCAAGATGCCTTTGAGTGTG	TGTGGTGAAGGTGTGAAGGAA
*ADRP*	TGGCAGCAGCAGTAGTGGA	ACATAAGCGGAGGACACAAGG
*MCP-1* promoter (ChIP analysis)	CACCCCATTACATCTCTTCCCC	TGTTTCCCTCTCACTTCACTCTGTC

### Immunoblotting

Total cell lysates for immunoblotting were collected as previously described [43], [44]. For detecting nuclear translocation of p65, nuclear extracts were prepared as previously described [43], [44]. Equal amounts of protein samples were resolved by sodium dodecyl sulfate-polyacrylamide gel electrophoresis and transferred to polyvinylidene fluoride membranes (Immobilon, Bedford, MA, USA). The membranes were incubated with the appropriate antibodies, washed and incubated with horseradish peroxidase-coupled secondary antibodies (Amersham, Buckinghamshire, UK). After washing, the blots were visualized by using an enhanced chemiluminescence detection system (Perkin Elmer Life Science, Boston, MA, USA).

### Small interference RNA transfections against *PPARδ*

Control siRNA and siRNA against *PPARδ* were purchased from Ambion (Austin, TX, USA). Transient transfections of siRNA were performed using Lipofectamin 2000 (Invitrogen, Carlsbad, CA, USA) [43], [44]. To determine the effect of siRNA against *PPARδ* on palmitate- and TNFα-induced *MCP-1* expression, all experiments were performed 24 h post-siRNA transfection.

### ChIP analysis

The ChIP experiment was performed using the ChIP-IT kit from Active Motif (Carlsbad, CA, USA) according to the manufacturer's instructions [44]. The primer sequences are listed in Table 1.

### Retroviral infection

pBABE-*PPARδ* vectors were obtained from Addgene. Retroviral infections were performed as previously described [43], [44]. Briefly, HEK293T cells were transfected with pBABE and pBABE-*PPARδ* using Lipofectamin 2000 reagent. Forty-eight hours post-transfection, the media containing retroviruses were collected, centrifuged, and transferred into the cultured proximal tubular cells, which were pre-treated with polybrene (1 µg/mL) for 30 min. Retrovirus-infected cells were selected by treatment with puromycin (2.5 µg/mL) for several days. We confirmed overexpression of PPARδ by immunoblotting and real-time PCR.

### Statistical analysis

Results are expressed as mean ± SEM. ANOVA with subsequent Scheffe test was used to determine the significance of differences in multiple comparisons. A *p* value <0.05 was considered statistically significant.
